# Fig Seeds as a Novel Oil Source: Investigating Lipochemodiversity Through Fatty Acids Profiling and FTIR Spectral Fingerprints

**DOI:** 10.3390/plants14060945

**Published:** 2025-03-17

**Authors:** Charaf Ed-dine Kassimi, Karim Houmanat, Ahmed Irchad, Rachid Aboutayeb, Abdessamad Ben Moumen, Aziz Fadlaoui, Ibtissame Guirrou, Fedoua Diai, Lhoussain Hajji, Lahcen Hssaini

**Affiliations:** 1 Agro-Food Technology and Quality Laboratory, Regional Center of Agricultural Research of Meknes, National Institute of Agricultural Research, Rabat 10090, Morocco; charafeddine.kassimi@inra.ma (C.E.-d.K.); k.houmanat@gmail.com (K.H.); aziz.fadlaoui@inra.ma (A.F.); ibtissame.guirrou@inra.ma (I.G.); 2 BioActives, Health and Environment Laboratory, Faculty of Science, University Moulay Ismail, P.O. Box 11201, Zitoune, Meknes 50100, Morocco; fe.diai@edu.umi.ac.ma (F.D.); i.hajji@umi.ac.ma (L.H.); 3 National Research Institute for Agriculture, Fisheries and Environment (INRAPE), Ex CEFADER, M’dé Ngazidja, Moroni P.O. Box 1406, Comoros; irchadahmed04@gmail.com; 4 Soil, Water, Plant Laboratory, Regional Center of Agricultural Research of Settat, National Institute of Agricultural Research, Avenue Ennasr, BP 415 Rabat Principale, Rabat 10090, Morocco; rachid.enginer@gmail.com; 5 Laboratory of Agricultural Production Improvement, Biotechnology and Environment, Faculty of Sciences, Mohammed I University, Oujda 60000, Morocco; abdenmoumen@gmail.com

**Keywords:** fig seed oil, *Ficus carica* L., lipochemodiversity, fatty acid composition, genotypic variation, oil fingerprints

## Abstract

Fig seeds (*Ficus carica* L.), a previously overlooked component of the fig fruit, have recently garnered attention as a valuable source of atypical vegetable oil and food-based ingredient. This study evaluated the oil content, fatty acid composition, and molecular FTIR-based signatures of 21 *Ficus carica* L. genotypes growing in an ex-situ collection. Gas chromatography analysis revealed high levels of linolenic acid (18.11 ± 0.255% to 42.276 ± 0.173%), followed by linoleic acid (27.75 ± 0.019% to 36.68 ± 0.046%). Palmitic acid (6.671 ± 0.006% to 8.908 ± 0.005%) and stearic acid (2.562 ± 0.009% to 4.160 ± 0.011) were the predominant saturated fatty acids (TSFA). The calculated oleic desaturation ratio (ODR), linoleic desaturation ratio (LDR), and ω6/ω3 ratio ranged from 0.466 ± 0.0284 to 0.710 ± 0.002, 0.330 ± 0.0998 to 0.595 ± 0.08, and 0.680 ± 0.283 to 2.025 ± 0.002, respectively. The desaturation efficiency from oleic to linoleic acid (ODR) was consistently lower than the desaturation from linoleic to linolenic acids (LDR) across all cultivars. ‘Aicha Moussa’ and ‘Amtala Arch’ exhibited the highest ODR and LDR (0.710 ± 0.002 and 0.595 ± 0.0779, respectively), potentially explaining the high C18:3 (linolenic acid) content in these cultivars. Notably, ‘Amtala Arch’ had an average linolenic acid content of 42.762 ± 0.173%. These findings highlight the significant lipochemodiversity within fig seeds, requiring further investigation into the potential for valorizing fig processing byproducts and creating new investment opportunities. FTIR-ATR spectroscopy coupled with chemometrics proved effective in characterizing molecular fingerprints, enabling both the rapid assessment of fig seed lipochemodiversity and enhanced sample authentication and classification.

## 1. Introduction

Figs (*Ficus carica* L.), one of the earliest fruits documented in written history [1], are a vital component of the Mediterranean diet and hold a significant place in global agriculture. As the world’s third-largest producer, the fig industry recognizes the importance of continued research and development for this species. In Morocco, figs are experiencing renewed interest for their potential to provide valuable raw material for the agro-industry and drive socio-economic development, particularly in remote or mountainous regions [2]. Therefore, figs are not only historical and nutritional assets but also a key contributor to the sustainable development of Morocco’s agricultural sector.

Despite the established significance of figs, there remains a notable gap in research regarding the value and potential of fig seeds. Although fig seeds are known to contribute to the overall quality of the fruit, they are often overlooked during processing [3]. This gap in research underscores the necessity for a deeper understanding of fig seeds’ composition and potential applications. Fig seeds, if valued within a circular economy framework, could nonetheless offer investment opportunities by moving away from the traditional linear model. The seeds could provide a new and atypical source of oil or serve as the basis for new nutraceuticals and innovative market formulations. Even though figs are not widely processed by industry, the quantities of seeds recoverable from losses generated during harvesting, processing, or packaging could be directed towards new forms of lipochemical valorization or used for nutraceutical purposes [3].

Recent studies have begun to explore the potential of fig seeds as a valuable source of atypical vegetable oil, highlighting their interesting lipid profiles and promising oil yields, which can reach up to 40%. Fig seeds, traditionally discarded during the processing of figs into syrup or jam, can be recovered and transformed into highly valuable oil, offering significant market profitability. Moreover, fig losses in the orchard and during or after harvesting, as well as figs with low marketability that cannot be sold, present additional opportunities for oil extraction. Even dried figs, typically destined for animal feed due to their low marketability and quality, can be repurposed for oil production. This repurposing aligns with sustainable agricultural practices by reducing waste and optimizing resource utilization [3].

The integration of fig seeds into a circular economy framework could offer investment opportunities, rather than adhering to the traditional linear model. The seeds could provide a new and atypical source of oil or serve as the basis for new nutraceuticals and innovative market formulations. Even though figs are not widely processed by industry, the quantities of seeds recoverable from losses generated during harvesting, processing, or packaging could be directed towards new forms of lipochemical valorization or used for nutraceutical purposes [3].

The seeds of the fig tree can generate a significant amount of waste, especially after the harvest. However, these seeds also offer great potential for revaluing within the circular economy. They could help reduce food waste and promote a sustainable resource management model by being transformed into useful products. Indeed, the integration of these wastes into valorization cycles would contribute to the reduction in environmental impacts and the optimization of available resources at the same time. In low-income countries, food losses are primarily due to managerial and technical limitations related to harvesting methods, storage, transportation, and processing. Major contributing factors include the lack of adequate refrigeration, infrastructure, and effective packaging and marketing systems. Meanwhile, for middle- and high-income countries, food waste is more closely related to consumer behavior, strict food safety policies, and quality standard requirements [4,5]. The estimation of food loss and waste varies across food categories, including fruits and veg, roots and tuber, grains, oilseeds, milk, meat, fish, and shellfish [4,5,6]. The FAO reports that the F&V and Roots and Tubers categories account for 44% and 20%, respectively, of total global food losses, meaning that fruits and vegetables account for 66% of total food losses by weight. Furthermore, the FAO reports that 45 to 55 per cent of the global production of F&Vs is lost or wasted along the supply chain [4,5,6]. A report by the NRDC found that 52% t of all fruit and vegetables produced in the United States, Canada and Australia are wasted, and that in New Zealand, fruit and vegetables are lost or wasted [7,8].

Previous research indicates that fig seed oil is typically pale yellow and exhibits considerable variability in oil content, with some genotypes yielding up to 30% oil [3]. Factors influencing fig seeds’ size and composition include oil content, include genetics, geographic location [9,10], and pollination methods [11]. The oil yield of fig seeds is comparable to that of pumpkin, honeydew, and mangos teen, and surpasses that of durian seeds (1.8%) [12], Opuntia ficus indica seeds (5.4 to 9.9%) [13], and guava seeds (16%) [14]. The high oil yield makes fig seeds a promising resource for industrial applications. Beyond industrial uses, fig seed oil also shows potential as a valuable ingredient in food products. It is a good source of plant-based polyunsaturated fatty acids and has been identified as a rich source of γ-tocopherol [15], which has garnered interest for its potential synergistic effects with α-tocopherol in promoting health benefits [16]. Despite these promising characteristics, fig seed oil remains relatively understudied, leaving many questions about its full potential unanswered. Today, despite the promising potential of fig seeds and their oil, research on their chemodiversity has been limited, with only four genotypes studied to date [3,4,5,6,7,8,9,10,11,12,13,14,15,16,17]. This work provides a basis for expanded research to fully exploit this valuable resource. Similar work has shown that FTIR-ATR spectroscopy can effectively differentiate and categorize fig genotypes [2,3,4,5,6,7,8,9,10,11,12,13,14,15,16,17]. Building upon these previous works, this study expands this analysis to a wider range of 21 fig genotypes, including aiming to provide a more comprehensive understanding of fig seed lipochemo diversity. 

The present work was designed to screen and evaluate the lipid profiles of different fig genotypes grown in an ex-situ collection, which may provide a deeper understanding of their nutritional and functional qualities, thereby informing future studies on fig oils and their food, health, and industrial applications.

## 2. Results and Discussion

### 2.1. Oil Content

The fig seed has the typical round and yellow appearance. According to Hssaini et al. [3], the average weight of one thousand seeds is approximately 1.14 ± 0.01 g. However, as shown in Table 1, the weight of seeds per fig fruit can vary considerably depending on the genotype. For example, the ‘Aicha Moussa’ genotype has a minimum seed weight of 1.39 ± 0.89 g per fruit, whereas the ‘El Qoti Lezreq’ genotype has a maximum seed weight of 2.46 ± 0.02 g per fruit. These findings are consistent with the range of 1.14 ± 0.35 to 2.45 ± 0.02 g/fruit reported by Irchad et al. [18].

The data presented in Table 1 reveal that the oil content of the fig seeds exhibits significant variation, ranging from 6.69 ± 0.14% to 39.97 ± 0.01%. This variation is evident across different varieties, with the ‘Bourqui’ variety displaying the highest oil content at 39.97 ± 0.01%, and the ‘INRA 2105’ variety showing the lowest oil content at 6.69 ± 0.14%. Irchad et al. [18] reported similar findings, noting that the ‘Bourqui’ variety has the highest oil content at 39.68%, while the ‘INRA 2105’ variety recorded the lowest oil content at 15.06%. The findings of Irchad et al. [14] closely align with our results for the ‘Bourqui’ variety but present a higher minimum value for the ‘INRA 2105’ variety compared to our lowest observed oil content for this variety.

Nakilcioğlu-Tas. [19] reported oil content in fig trees ranging from 23.06% to 23.67%. This range falls within the mid-spectrum of our observed values, suggesting a moderate oil content for the varieties studied compared to the broader range found in our study. Naoui et al. [20] found that the maximum oil content observed was 7.25% in their study. This value is significantly lower than the highest values reported both in our study and by Irchad et al. [18], indicating substantial differences in oil content based on geographic or varietal factors. Hssaini et al. [4] studied four cultivars in Morocco and reported a maximum oil content of 29.65% and a minimum of 21.54%. These values are consistent with a subset of our findings, particularly for varieties with mid-range oil content, and highlight the variability within fig tree varieties even within a specific region.

Furthermore, Hssaini et al. [11] revealed the critical role of pollination in determining the oil content of fig trees. Their research demonstrated that pollination significantly impacts oil production, with yields typically ranging from 25.93% to 32.59%. These values align well with the range observed in our study, further supporting the validity of our findings. In conclusion, our results corroborate the existing literature, underscoring the substantial influence of varietal differences on the oil content of fig seeds. The observed variations can be attributed to multiple factors, including the genetic makeup of each variety, environmental conditions, and agricultural practices. 

These findings emphasize the importance of considering varietal selection, geographic factors, and pollination practices to optimize oil yield and content in fig tree cultivation

### 2.2. Fatty Acids Composition of Seeds Oil

**Figure 1:**
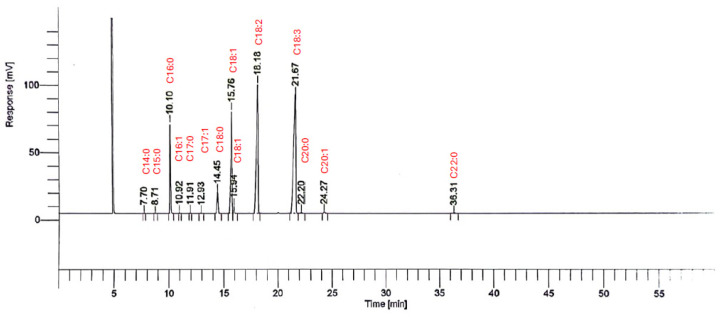
Typical GC chromatogram of the fatty acids of fig seed oil. Example of variety ‘Hayoul ’ (C14:0: myristic acid; C15:0: pentadecylic acid; C16:0: palmitic acid; C16:1: palmitoleic acid; C17:0: margaric acid; C17:1: heptadecenoic acid; C18:0: stearic acid; C18:1: oleic acid; C18:2: linoleic acid; C18:3: alpha-linolenic acid; C20:0: arachidic acid; C20:1: eicosenoic acid; C22:0: behenic acid.

The identification of the fatty acids of fig seed oil across all cultivars reveled a total of 13 fatty acids (Figure 1). As mentioned in Table 2, TSFA were mainly composed of myristic (C14:0), pentadecylic (C15:0), palmitic (C16:0), margaric (C17:0), stearic (C18:0), arachidic (C20: 0), and behenic (C22:0) acids. Among these, palmitic acid was the predominant one, with an average of 7.806 ± 0.033, followed by stearic acid, with an average of 3.336 ± 0.014. They were evaluated at lower levels, whereas myristic (C14:0), pentadecylic (C15:0), margaric (C17:0), arachidic (C20:0), and behenic acid (C22:0) were observed at trace amounts (0.029 ± 0.013, 0.025 ± 0.001, 0.063 ± 0.003, 1.091 ± 0.1222, 0.091 ± 0.010). These results are in agreement with those reported by Hssaini et al. [3] showing that palmitic acid (C16:0) and stearic acid (C18:0) were the dominant fatty acids with an average of 8.75 ± 0.21 and 3.336 ± 0.014, respectively, while arachidic (C20:0), margaric (C17:0), and pentadecylic (C15:0) acids presented lower levels (0.09 ± 0.04, 0.046 ± 0.01 and 0.02 ± 0.03%).

These values are also in accordance with those reported for ‘Sarilop’ seeds Nakilcioğlu-Taş [19] and fit with the oil composition of other fruit seeds such as guava (87.3% TUFA and 11.8% TSFA) [21] and Opuntia ficus-indica (83% TUFA and 16-17% TSFA) [13]. In another study at Algeria, seven types of (*Ficus carica* L.) fruits from Algeria were investigated; the results show that palmitic acid was the major saturated fatty acid with values ranging from 8.17 to 34.96% and both capric and lauric acids were detected in lower amounts with values ranging from 0.03 to 0.12% and 0.05 to 0.52% [16]. In our samples, linolenic acid (C18:3) was the most abundant fatty acid, ranging from 42.76 ± 0.173% in ‘Amtalaa Arch’ to 18.17 ± 0.2554% in ‘Aicha Moussa’, followed by linoleic acid (C18:2), which varied from 36.51 ± 0.018% in ‘Melissosyki’ to 27.75 ± 0.019% in ‘INRA 2603’. Oleic acid (C18:1) was present in amounts ranging from 20.23 ± 0.035% in ‘Breval Blanca’ to 7.24 ± 0.012% in ’Breba Blanca’. Table 2 summarizes the concentration ranges of other identified fatty acids across the studied cultivars. Notably, arachidic acid (C20:0) ranged from 18.17 ± 25.37% in ‘Aicha Moussa 2208’ to 0.18 ± 0.005% in ‘Rhoul’, while palmitic acid (C16:0) was found in amounts between 8.9 ± 0.005% in ‘Melissosyki’ and 6.67 ± 0.006% in ‘Rhoul’. Stearic acid (C18:0) ranged from 4.16 ± 0.01% in ‘INRA 2603’ to 2.56 ± 0.009% in ‘Rhoul’. Eicosenoic acid (C20:1) varied from 0.3 ± 0.00% in ‘Breval Blanca’ to 0.13 ± 0.015% in ‘White Adriatic’, and behenic acid (C22:0) ranged from 0.17 ± 0.013% in ‘Aicha Moussa’ to 0.07 ± 0.003% in ‘Bourqui’. Myristic acid (C14:0) was found in amounts between 0.12 ± 0.014% in ‘Aicha Moussa 2208’ and 0.017 ± 0.00% in ‘Bourjassate Noire’. Margaric acid (C17:0) ranged from 0.08 ± 0.01% in ‘Breba Blanca’ to 0.05 ± 0.001% in ‘Amtalaa Arch’, while palmitoleic acid (C16:1) varied from 0.07 ± 0.00% in ‘Amtalaa Arch’ to 0.044 ± 0.00% in ‘Aicha Moussa’. Finally, pentadecylic acid (C15:0) was detected in amounts ranging from 0.03 ± 0.00% (JUA) to 0.017 ± 0.00% in ‘Amtalaa Arch 2210’.The notable presence of unsaturated fatty acids (UFA) such as linolenic (C18:3), linoleic (C18:2), and oleic acid (C18:1) in the fig seed oils analyzed is important because of their recognized health advantages (Figure 1). Having a higher intake of UFA is essential for keeping the immune response in check and providing defense against different health issues, such as heart disease [22,23].

Alpha-linolenic acid (ALA, C18:3) is crucial in reducing the severity of inflammatory diseases [24]. Likewise, linoleic acid (LA, C18:2) plays a role in various bodily functions, such as enhancing the immune response and aiding in blood clotting by being transformed into eicosanoid hormones [25]. In addition, oleic acid (OA, C18:1) is known for its effectiveness in reducing levels of cholesterol in the blood [26,27].

Studies have shown that OA can lower LDL cholesterol levels and maintain or even raise HDL cholesterol levels, thereby lowering the risk of heart disease [28,29]. Aside from reducing cholesterol, the anti-inflammatory properties of OA may also enhance its heart-protecting advantages [26].

### 2.3. Fatty Acids Ratios

The comparison of individual fatty acids between different fig cultivars may be related to the need for pollination for seed development. Typically, seeds remain veined without pollination. As a result, various ratios such as the oleic desaturation ratio (ODR), linoleic desaturation ratio (LDR), and omega-6 to omega-3 ratio (ω-6/ω-3) have been employed. These ratios are often used to emphasize other types of seed oil and to reduce the effects of breeding change [30].

The LDR varied significantly between 0.330 ± 0.0998 for ‘Aicha Moussa 2208’ and 0.595 ± 0.0779 for ‘Amtala Arch 2210’, while the ODR ranged from 0.466 ± 0.0284 for ‘Amtala Arch’to 0.710 ± 0.002 for ‘Aicha Moussa’. The ω-6/ω-3 ratio fluctuated between 0.680 ± 0.0283 for ‘Amtala Arch’and 2.025 ± 0.002 for ‘Aicha Moussa’ among samples. The efficiency of the desaturation process from oleic acid to linoleic acid (ODR) was higher across all cultivars compared to the desaturation from linoleic to linolenic acids (LDR) (Table 3).

(1) $$\mathrm{ODR}\;(\mathrm{oleic}\;\mathrm{desaturation}\;\mathrm{ratio})=\frac{\%C18:1+C18:2\;}{\%\;C18:1+\%\;C18:2+C18:3}$$

(2) $$\mathrm{LDR}\;(\mathrm{linoleic}\;\mathrm{desaturation}\;\mathrm{ratio})=\;\frac{\%C18:3\;}{\;\%\;C18:2+\%C18:3}$$

The cultivars ‘Aicha Moussa’ and ‘Amtala Arch’ exhibited the highest ODR and LDR value, measuring 0.710 ± 0.002 and 0.595 ± 0.0779, respectively. This increased efficiency in the desaturation pathway explains the substantial increase in 18:3 content across all cultivars. Specifically, in ‘Amtala Arch’, the combination of high ODR and LDR resulted in a notably high C18:3 content, averaging 42.762 ± 0.173%.

The balance between omega-6 (ω-6) and omega-3 (ω-3) fatty acids, represented by linoleic acid and linolenic acid, respectively, is a critical determinant of oil quality and plays a significant role in human health [31]. In the study by Hssaini et al. [3], all samples exhibited a very low ω-6/ω-3 ratio, with an average value of 0.879. In contrast, blackberry seed oil has a ω-6/ω-3 ratio of approximately 3.63, which is three times higher than that found in elderberry seed oil (1.19). Nutritional societies emphasize the importance of maintaining a lower ω-6/ω-3 ratio for a healthier diet [32]. The German Nutrition Society recommends a lower ω-6/ω-3 ratio to support optimal health. The World Health Organization (WHO) and Food and Agriculture Organization (FAO) suggest that the ω-6/ω-3 ratio should be less than 5 [33,34]. This recommendation is based on evidence that a balanced intake of these essential fatty acids can help reduce the risk of inflammatory and chronic diseases.

For the local genotype ‘Aicha Moussa’, this ratio was 2.025, while it was 1.131 for the cultivar ‘Melissosyki’. However, the ω-6/ω-3 ratio was very low in the varieties ‘Amtala Arch’ and ‘Adroulaniki’, for which the recorded values were 0.680 ± 0.0283 and 0.699 ± 0.0769, respectively. Several reports have shown that the biological availability and activity of omega-6 fatty acids are inversely linked to the concentration of omega-3 fatty acids in plant tissues [31]. Indeed, the healthy ω-6/ω-3 ratio should be lower than 5 [29]; it has been recommended that the consumers evolve on a diet with a ratio of ω-6 to ω-3 of ~1 [24]. A lack of ω-3 FA dietary intake causes a high ratio in our daily diet, and has been linked to some health issue such as blood lipid, autoimmune, cardiovascular, and inflammatory diseases [35].

### 2.4. Assessing Lipochemodiversity Through ANOVA

The analysis of variance (ANOVA one-way) revealed significant diversity within the fig seed collection, with oil content and most fatty acid variables and related ratios exhibiting statistically significant differences among the 21 (*Ficus carica* L.) genotypes (*p* < 0.05) Table 4.

The desaturation pathway, reflected in the linoleic desaturation ratio (LDR; F = 16,088.53, *p* ≈ 0) and oleic desaturation ratio (ODR; F = 6978.21, *p* ≈ 0), showed particularly strong variations among genotypes. Similarly, significant differences were observed in the levels of key unsaturated fatty acids, including oleic acid (C18:1; F = 7458.75, *p* ≈ 0) and linoleic acid (C18:2; F = 5879.89, *p* ≈ 0), contributing to the overall variation in monounsaturated fatty acid (MUFA) content (F = 5610.33, *p* ≈ 0). While less pronounced, statistically significant differences were also detected for some minor fatty acids, such as C20:0, C14:0, and C17:1 (*p* < 0.05). Unlike other fatty acid metrics, the omega-6 to omega-3 (ω6/ω3) ratio showed no significant variation across genotypes (F = 1.005, *p* = 0.477), indicating a stable balance of these essential fatty acids. This consistency suggests that fig seeds could serve as a reliable source of oils with favorable ω6/ω3 ratios, which are critical for reducing inflammation and promoting cardiovascular health. These results confirm that fig varieties possess distinct fatty acid profiles, offering a range of compositional variations that could be strategically leveraged for specific nutritional or industrial applications.

### 2.5. Characteristics of FTIR-ATR Fingerprints

**Figure 2:**
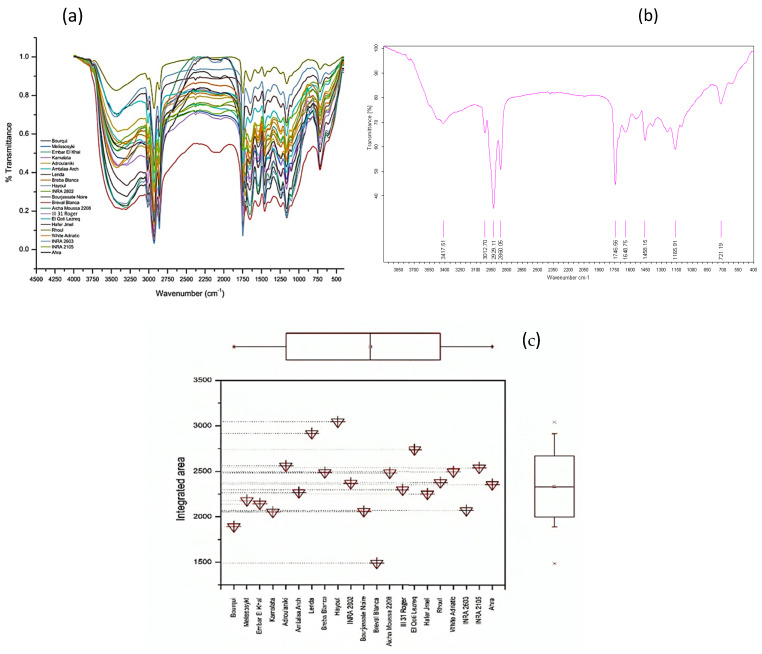
(**a**) FTIR-ATR spectroscopy data of investigated fig seeds samples in the wavenumber range from 4000 to 450 cm^−1^. (**b**) Boxplot illustrating the total integrated area of the entire FTIR-ATR spectrum for each fig seed cultivar to show the differences in vibration intensities across the samples. (**c**) FTIR-ATR spectrum of the local clone ‘El Qoti Lezreq’ fig seeds highlighting key peaks in the wavenumber range of 4000 to 450 cm^−1^.

To gain insights into the molecular composition of fig seeds, we utilized FTIR-ATR. This technique is renowned for its ability to provide detailed molecular fingerprints, making it ideal for distinguishing subtle differences in biological samples [13,36,37,38]. In this study, FTIR-ATR was utilized to examine the molecular fingerprints in fig seeds and assess their phenotypic diversity by scanning the samples in the wavenumber range of 4000 to 450 cm^−1^ with a spectral resolution of 4 cm^−1^. The spectra of the 21 fig seed varieties are shown in Figure 2a. As expected, the spectra of all the seeds appear similar. However, analysis using chemometric tools reveals significant differences, particularly by evaluating the integrated intensities of each major vibrational region. These differences, although sometimes small, indicate molecular differences between the samples. Overall, peaks at different wavenumbers were identified in each spectrum and assigned to specific functional groups [36], as summarized in Table 5. This table provides a detailed assignment of the bands, allowing for the identification of 13 IR signatures (Figure 2b). Specifically, FTIR spectra in the Figure 2b showcases of vibrations and major peaks of the genotype ‘El Qoti Lezreq’ within the wave number range of 4000 to 450 cm^−1^.

While Table 5 provides a comprehensive overview of identified wavenumbers and their assignments, this analysis will delve into some spectral features. The absorption at ~1648 cm⁻^1^ is most probably ascribed to C=C stretching vibrations from unsaturated fatty acids [39,40]. This assignment aligns with the documented high content of polyunsaturated fatty acids in *Ficus carica* L. seed oil, particularly linoleic acid, which constitutes 78–88% of the total fatty acid profile [17,18,19,20,21,22,23,24,25,26,27,28,29,30,31,32,33,34,35,36,37,38,39,40,41].

The band at 1544 cm⁻^1^ is likely associated to the C=C stretching of aromatic rings from minor bioactive components such as phenolic compounds [17]. This interpretation is supported by studies identifying various phenolic constituents in fig seed extracts despite the highly non-polar nature of hexane as an extraction solvent [3,42]

The distinctive peak at 721 cm⁻^1^ is unequivocally assigned to the CH_2_ rocking vibrations (-(CH_2_)n-) characteristic of long-chain fatty acids [43,44]. This band serves as a definitive marker for lipid structures and is consistently observed in the fingerprint region of various vegetable and seed oils [45,46]. The intensity of this peak correlates with the methylene chain length in fatty acids, providing valuable information about the structural composition of the extracted oil [47].

**Table 5:** FTIR peaks assignments for functional groups found in fig (*Ficus carica* L.) seeds.

Wavenumber (cm^−1^)	Functional Groups	Modes of Vibration	Assignments	References
3417	δ(O–H)	Stretching vibration	Intramolecular hydrogen bond between C(3)OH• • •O(5) and C(6)O• • •O(2)H	[36,48,49]
3071	ν(=C–H)	Bending vibration	Unsaturated fatty acids	[49,50,51]
3012	δ(C–H)	Stretching vibration	Unsaturated fatty acids particularly present in oleic acid or linoleic acid	[49]
2929 2860	δ(C–H), δ(–CH2–)	Stretching vibration	Methylene and methyl groups (lipids)	[50,51,52]
1746	δ(C=O-H), δ(O=C-H)	Stretching vibration	Ester carbonyl functional group of the triglycerides and fatty acids	[38,40,41,42,43,44,45,46,47,48,49,50,51,52,53]
1648	δ(C=C)	Stretching vibration	Saturated fatty acids	[39,40]
1544	δ(C=C)	Stretching vibration	Aromatric rings from minor bioactive compounds (i.e., phenols)	[17]
1458	δ(C–H), ν(C–H)	Stretching and bending vibrations	Lipids and cholesterol esters	[40,53,54]
1386 1239	ν(C–H), δ(C–O)	Stretching and bending vibrations	Esters	[39,50,51,52,53,54,55]
1165	δ(C–O), ν(C–C)	Bending vibration	Esters	[50,51,52,53]
1102	δ(C–O)	Stretching vibration	Unsaturated esters and esters derived from secondary alcohols	[39]
721	-(CH_2_)n-	Rocking	Long chain fatty acids	[43,44]
611	ν(–(CH2)_n_–), δ(–HC=CH–(*cis*-))	Bending vibration (rocking)	Disubstituted olefinic *cis*-alkenes	[40,41,42,43,44,45,46,47,48,49,50,51,52,53,54,55,56]

The main peaks with their respective assignments are summarized in Table 5, revealing the unique composition of fig seed oils as typical molecular signatures. It is important to acknowledge that while the fig seed spectra exhibit general similarities and overlap in Figure 2c, subtle yet significant variations exist. These variations manifest as differences in the integrated intensity of specific bands and the precise frequencies at which maximum absorption occurs. Such spectral nuances are not unexpected, as they likely reflect inherent genotypic variations among the fig seed cultivars. These variations can result in differences in the relative abundance and composition of key biomolecules, ultimately influencing the spectral fingerprints observed. Therefore, these subtle spectral differences hold valuable information and can be further explored using chemometric approaches to discriminate between fig seed cultivars based on their unique biochemical profiles.

The box plot presented in Figure 2a effectively visualizes the phenotypic diversity among fig seed varieties, as reflected in their vibrational intensities within the 3500–1000 cm⁻^1^ spectral region. This visualization, which depicts the distribution of integrated intensities for each genotype, provides insights into the median, interquartile range, and potential outliers. A one-way analysis of variance (ANOVA) was conducted on the integrated intensities of the key peaks, which revealed statistically significant differences among the fig seed samples (*p* < 0.05). This finding lends support to the notion that genotypic variation has an impact on the overall molecular profiles of the seeds. For example, the ‘Hafer Jmel’ genotype exhibited an integrated area of approximately 2250, which was significantly different from the area displayed by the White Adriatic genotype, which was approximately 2500. It is noteworthy that the ‘Breval Blanca’ genotype exhibited the lowest integrated area, approximately 1500, which significantly differentiates it from the other cultivars examined. These findings highlight the significant impact of genotype on the lipochemical profiles of fig seeds. Although the observed differences in integrated intensities may appear numerically subtle, they are likely indicative of variations in the relative abundance and composition of key nutritional components. This is consistent with existing research indicating that genotype affects the quality of fig seeds, including but not limited to their nutritional content. This study demonstrates the value of FTIR-ATR spectroscopy as a rapid and non-destructive method for analyzing the characteristics of fig seeds. The capacity to discern subtle variations in spectral fingerprints associated with different genotypes positions this technique as a potential asset in breeding programs. Such programs, informed by these spectral analyses, could focus on optimizing fig seed quality and nutritional value for human consumption.

### 2.6. Principal Component Analysis

**Figure 3:**
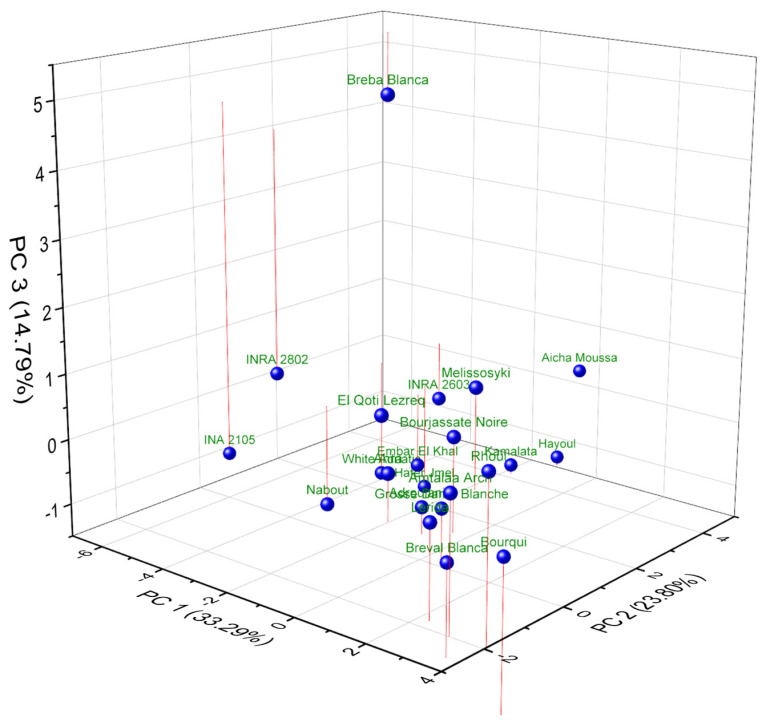
3D PCA plot of FTIR spectral fingerprints of studied fig seeds visualizing biochemical variation in fig seeds.

**Figure 4:**
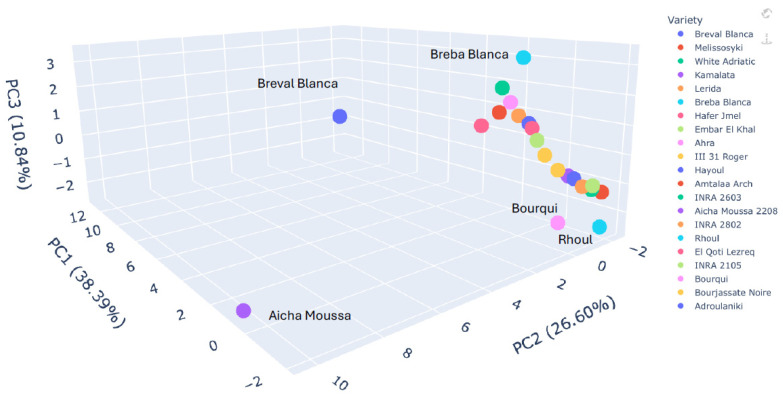
Lipochemical diversity of fig seed cultivars: a 3D PCA visualization based on fatty acid profiles.

Comparing the 3D PCA plots derived from FTIR spectral data (Figure 3) and lipid profiles (Figure 4) provides a multifaceted perspective on the lipochemodiversity within the fig seed collection. While both techniques contribute valuable insights, the lipid profile-based PCA demonstrates a clearer separation of genotypes, highlighting the nuanced variations in fatty acid composition that drive distinctions in oil quality and nutritional value. Conversely, the FTIR-based PCA, though sensitive to overall biochemical composition, reveals almost comparable general clustering, with the more distinct and slightly different classification of individuals, which is somehow evident. Interestingly, for some cultivars, they were tightly clustered and distinctly separated along PC1, particularly ‘Breba Blanca’, ‘Breval Blanca’, and ‘Aicha Moussa’. This classification suggests that FTIR spectroscopy can capture the subtle differences in fatty acid profiles that significantly influence oil characteristics. The dominance of PC1 (31.97%) in the FTIR-based PCA further supports this interpretation, implying that a single major source of variation, potentially related to lipids known to influence FTIR spectra, is primarily responsible for the observed separation.

On the other hand, the PCA plot constructed from lipid profiles exhibits a more pronounced separation of genotypes across all three principal components, underlining the significance of fatty acid composition and desaturation ratios as key drivers of lipochemotypic diversity. The cultivars ‘Breba Blanca’ and ‘Aicha Moussa 2208’ again occupied a distinct position on the PC1 axis, reflecting their unique fatty acid profiles rich in oleic acid (C18:1) and characterized by a low ω-6/ω-3 ratio. This clear separation extends to other genotypes as well. For example, ‘Bourqui’, ‘Rhoul’, and ‘Aicha moussa’ are clearly distinguished along PC2, primarily driven by variations in the relative abundance of long-chain saturated fatty acids (C14:0, C20:0, C22:0) and linolenic acid (C18:3), as reported in Table 6.

A comparison between the two PCA plots underscores the complementary nature of FTIR and lipid profiling for characterizing fig seed lipochemodiversity. FTIR offers a rapid and broad assessment of biochemical composition, but lipid profiling provides the crucial granularity needed to understand the nuanced variations in fatty acid profiles that directly impact oil quality and nutritional attributes. This interpretation is further reinforced by the variable loadings table, which reveals the dominant influence of unsaturated fatty acids (C18:1, C18:2, and C18:3) and their related ratios (ODR and LDR) on the separation observed in the lipid profile PCA. Conversely, the FTIR-based PCA is likely influenced by a broader range of biochemical components, including carbohydrates and proteins, explaining the less pronounced separation and the loading of minor fatty acids on PC2 and PC3. In conclusion, the comparative analysis of the two PCA plots reveals the power of a multifaceted approach to understanding fig seed lipochemodiversity. While FTIR provides a valuable initial assessment, lipid profiling adds a critical layer of detail, allowing for a more informed selection of genotypes for specific oil production and nutritional applications.

## 3. Materials and Methods

### 3.1. Plant Material and Experimental Design

Twenty-one different fig tree genotypes (*Ficus carica* L.) were selected from an ex-situ collection in the experimental station of the National Institute for Agricultural Research (INRA) in Meknes, Morocco. The trees, aged 16 years, and pruned to a cup shape, were systematically planted in a randomized complete block design, with each genotype represented by three trees spaced 5 m × 3 m apart. The collection included a wide range of fig genotypes, both exotic and local clone, as shown in Table 7.

### 3.2. Fruit Sampling

Figs were harvested during the summer at fully ripeness. Genotypes were chosen based on their size and seeds number. Full ripeness was determined by the presence of a reddish-purple color covering three quarters of the fruit, indicating that the fruit could be easily detached from the branch. To ensure representative sampling, fruits were randomly selected from various parts of the canopy at a height of 160 cm.

### 3.3. Seed and Oil Extraction

Fig seeds were manually separated from the pulp using a 10% ethanol solution. The mixture was stirred for approximately 10 min and then allowed to settle, facilitating the separation of the seeds, which floated to the surface. The collected yellow, round seeds were thoroughly washed with distilled water and were air-dried at room temperature for 24 h. Each batch of dried seeds was then finely ground using an IKA A11 basic grinder to achieve a powdered consistency. Oil extraction was performed using a Soxhlet apparatus. Twenty grams of the powdered seeds was mixed with 150 mL of *n*-hexane in cellulose cartridges. Extraction continued for up to 4 h, and the solvent was then removed by evaporation using a Buchi rotavapor R-200 set at 40 °C. Oil weight was determined following the methodology described by Chougui et al. [23]:

Oil yield (%) = [(M1 − M0)/M2] × 100 (3)

where

M0 is the weight of the empty flask in grams (g);

M1 is the weight of the flask after evaporation of the solvent (g);

M2 is the weight of the seed powder (g).

The average weight of the oil cake was also calculated. Extracted oils were stored in dark glass bottles at 4 °C until ready for analysis.

### 3.4. Seed Oil Analysis

#### 3.4.1. Chemical Inputs

All chemicals used were of analytical grade and obtained from Sigma–Aldrich (Saint Louis, MO, USA).

#### 3.4.2. Fatty Acids Profiling

Fatty acid composition was determined following the official EU standard methods described in Annexes II and IX of European Community Regulation EEC/2568/91. Briefly, methyl esters (FAME) were prepared by a cold alkaline transesterification using methanolic potassium hydroxide, followed by extraction with *n*-heptane. Gas chromatography (GC) was performed using a Varian CP 3380 chromatograph equipped with a capillary column (CP-Wax 52 CB: length = 30 m, diameter = 0.25 mm, film thickness = 0.20 mm), a split–splitless injector with a CP-8400 autosampler, and an FID detector. The injector, detector, and oven were set at temperatures of 220 °C, 230 °C, and 180 °C, respectively. Hydrogen was used as the carrier gas at an internal pressure of 110 k Pa. The oven temperature program started at 70 °C for 4 min, increased to 110 °C at a rate of 8 °C/min, further increased to 170 °C at 5 °C/min and held for 10 min, and finally reached 250 °C at 4 °C/min and held for a further 15 min. A 1 µL sample was injected at a split ratio of 1:50. Results were expressed as relative percentages of individual fatty acids determined by internal normalization of chromatographic peak areas. A reference standard mixture (C4-C24, FAME Mix 37) was used for calibration and identification of FAME based on retention times.

#### 3.4.3. FTIR-ATR Spectroscopy

FTIR spectroscopic analysis was performed on unmethylated fig seeds oil using Bruker Vertex 70 FTIR spectrometer at wavelengths from 4000 to 450 cm^−1^ with a spectral resolution of 4 cm^−1^. Measurements were carried out under standard room temperature conditions, using a volume of 50 µL of pure oil averaging eight replicates per FTIR spectrum. Each spectrum resulted from the accumulation of 128 scans. Prior to analysis, a background spectrum was obtained from an empty germanium crystal surface and subtracted from the sample spectra. The crystal cell was thoroughly cleaned with ethyl alcohol and warm water between scans and then dried with absorbent paper. The acquired spectra were then processed using specialized Origin Lab 9 pro software to obtain absorbance values for subsequent analysis [13].

To prepare the FTIR spectral data for robust analysis, adjustments were made for baseline, transmission, and ATR effects. The ATR correction involved using a 45-degree angle, one reflection, and a germanium crystal with a refractive index of 1.5, interaction depth of 50 µm, and surface area of 1.8 mm^2^ [13].

To improve spectral resolution even more, a second derivative transformation was performed with a Savitzky–Golay filter (third-order polynomial, 11-point window), showing delicate spectral changes [7]. The initial analysis involved examining lipochemical data, such as oil content, fatty acid profiles, and desaturation ratios, using one-way ANOVA to evaluate lipochemodiversity. PCA was then separately used on the pre-processed FTIR spectra and the lipochemical data to assess the effectiveness of each method in distinguishing fig genotypes.

## 4. Conclusions

Fig seeds, long overlooked despite their potential as a valuable source of lipids and nutraceuticals, have emerged as a promising investment opportunity based on their high yield rate (up to 40%) and remarkable lipochemical diversity. The analysis revealed high concentrations of essential polyunsaturated fatty acids, with linolenic acid (18.11 ± 0.255% to 42.276 ± 0.173%) and linoleic acid (27.75 ± 0.019% to 36.68 ± 0.046%) predominating, alongside important saturated fatty acids including palmitic (6.671 ± 0.006% to 8.908 ± 0.005%) and stearic acid (2.562 ± 0.009% to 4.160 ± 0.011%). The oleic desaturation ratio (ODR), linoleic desaturation ratio (LDR), and ω6/ω3 ratio revealed significant genotype-dependent variations in lipid metabolism, with ‘Aicha Moussa’ and ‘Amtala Arch’ exhibiting superior desaturation efficiency—particularly ‘Amtala Arch’ with its exceptional linolenic acid content of 42.762 ± 0.173%. These variations, validated through ANOVA and PCA, highlight the profound influence of genetic factors on oil quality and yield. FTIR-ATR spectroscopy combined with chemometric analysis provided precise molecular fingerprinting, enabling efficient sample classification and establishing a robust analytical framework for future development. As the world’s third-largest fig producer, Morocco is strategically positioned to implement industrial-scale extraction of these oils from processing by-products, aligning with circular economy principles while creating high-value commodities. Future efforts should prioritize oil stability assessment and strategic breeding programs to enhance specific fatty acid profiles for targeted applications, fostering partnerships that maximize both economic returns and environmental sustainability in the expanding plant-based oil market.

## Figures and Tables

**Table 1:** Seed yield and oil content of different fig (*Ficus carica* L.) genotypes.

Genotypes	Seeds Yield (g/Fruit)	Seed Yield in (g/kg)	Oil Yield (%)
Breval Blanca	2.04 ± 0.3	117.95 ± 2.89	31.24 ± 0.09
Melissosyki	1.97 ± 0.48	114.81 ± 3.17	30.89 ± 0.14
White Adriatic	2.27 ± 0.93	89.03 ± 1.48	22.49 ± 0.42
Kamalata	2.10 ± 0.14	112.02 ± 2.11	28.87 ± 0.40
Lerida	2.25 ± 0.38	92.07 ± 2.92	30.28 ± 0.12
Breba Blanca	2.30 ± 0.18	78.68 ± 2.85	23.75 ± 0.28
Hafer Jmel	2.23 ± 0.4	107.23 ± 2.19	31.50 ± 0.81
Embar El Khal	2.09 ± 0.75	107.17 ± 3.18	30.77 ± 0.26
Ahra 2870	2.24 ± 0.05	101.10 ± 2.48	30.75 ± 0.44
III 31 Roger	1.93 ± 0.40	120.52 ± 1.22	29.18 ± 0.30
Hayoul 2265	2.18 ± 0.87	104.54 ± 2.55	28.67 ± 0.40
Amtalaa Arch	2.08 ± 0.12	116.87 ± 1.86	30.20 ± 0.0
INRA 2603	1.99 ± 0.55	109.35 ± 1.70	23.29 ± 0.15
Aicha Moussa	1.39 ± 0.89	107.75 ± 1.81	31.08 ± 0.70
INRA 2802	1.79 ± 0.2	131.62 ± 1.37	16.68 ± 0.69
Rhoul 2216	2.07 ± 0.78	110.46 ± 3.75	31.86 ± 0.13
El Qoti Lezreq	2.46 ± 0.02	58.18 ± 1.00	28.97 ± 0.55
INRA 2105	1.96 ± 0.45	121.95 ± 2.41	6.69 ± 0.14
Bourqui	1.39 ± 0.54	111.23 ± 2.52	39.97 ± 0.01
Bourjassate Noire	1.99 ± 0.51	114.72 ± 3.50	30.89 ± 0.42
Adroulaniki	1.95 ± 0.48	128.23 ± 2.98	26.54 ± 0.91

**Table 2:** Fatty acids profiles of fig seed oil determined by gas liquid chromatography (% *w*/*w*).

Cultivars	C14:0	C15:0	C16:0	C16:1	C17:0	C17:1	C18:0	C18:1	C18:2	C18:3	C20:0	C20:1	C22:0	MUFA	PUFA	TSFA	TUFA	MUFA/PUFA	TSFA/TUFA
Breval Blanca	0.021 ± 0.001	0.028 ± 0.003	8.145 ± 0.007	0.060 ± 0.000	0.063 ± 0.004	0.230 ± 0.0269	3.325 ± 0.007	20.235 ± 0.035	31.670 ± 0.001	35.785 ± 0.007	0.230 ± 0.001	0.300 ± 0.001	0.086 ± 0.001	5.206 ± 0.076	29.230 ± 0.014	1.7 ± 0.0031	14.7133 ± 0.0518	0.1781 ± 0.05375	0.1156 ± 0.0603
Melissosyki	0.023 ± 0.001	0.031 ± 0.001	8.908 ± 0.005	0.067 ± 0.001	0.069 ± 0.005	0.047 ± 0.001	3.643 ± 0.003	8.925 ± 0.023	36.518 ± 0.018	32.286 ± 0.052	0.260 ± 0.002	0.259 ± 0.000	0.089 ± 0.003	2.324 ± 0.071	25.909 ± 0.031	1.86007 ± 0.002727	13.0167 ± 0.0158	0.0897 ± 0.002303	0.14383 ± 0.00173
White Adriatic	0.040 ± 0.003	0.020 ± 0.004	7.834 ± 0.106	0.074 ± 0.005	0.060 ± 0.001	0.038 ± 0.001	3.312 ± 0.046	8.675 ± 0.100	29.417 ± 0.170	41.292 ± 0.231	0.239 ± 0.004	0.131 ± 0.0151	0.087 ± 0.004	2.229 ± 0.064	26.461 ± 0.167	1.656 ± 0.028	13.270 ± 0.110	0.084 ± 0.00384	0.1257 ± 0.002552
Kamalata	0.018 ± 0.001	0.021 ± 0.001	7.104 ± 0.030	0.064 ± 0.004	0.061 ± 0.006	0.044 ± 0.001	3.179 ± 0.004	8.849 ± 0.099	31.634 ± 0.033	39.607 ± 0.209	0.237 ± 0.000	0.254 ± 0.000	0.081 ± 0.001	2.303 ± 0.026	26.697 ± 0.113	1.528 ± 0.006	13.408 ± 0.0573	0.0862 ± 0.00228	0.114 ± 0.00105
Lerida	0.020 ± 0.000	0.025 ± 0.001	8.272 ± 0.022	0.058 ± 0.001	0.065 ± 0.002	0.042 ± 0.001	3.337 ± 0.013	10.237 ± 0.038	30.671 ± 0.006	36.421 ± 0.102	0.244 ± 0.02	0.287 ± 0.001	0.080 ± 0.008	2.656 ± 0.010	25.776 ± 0.049	1.720 ± 0.007	12.952 ± 0.0248	0.1030 ± 0.00211	0.133 ± 0.00275
Breba Blanca	0.023 ± 0.001	0.039 ± 0.001	7.883 ± 0.030	0.051 ± 0.001	0.082 ± 0.001	0.037 ± 0.001	3.670 ± 0.006	7.247 ± 0.012	34.741 ± 0.047	38.369 ± 0.053	0.269 ± 0.011	0.255 ± 0.001	0.089 ± 0.002	1.897 ± 0.004	26.785 ± 0.037	1.722 ± 0.007	13.449 ± 0.0189	0.071 ± 0.0094	0.1280 ± 0.00373
Hafer Jmel	0.084 ± 0.0092	0.031 ± 0.011	7.411 ± 0.194	0.070 ± 0.016	0.059 ± 0.011	0.040 ± 0.005	3.870 ± 0.062	12.534 ± 0.054	30.639 ± 0.245	32.102 ± 0.259	0.147 ± 0.0170	0.275 ± 0.001	0.085 ± 0.003	3.229 ± 0.019	25.092 ± 0.186	1.669 ± 0.077	12.610 ± 0.0967	0.1287 ± 0.00104	0.1324 ± 0.008017
Embar El Khal	0.021 ± 0.001	0.027 ± 0.001	7.518 ± 0.000	0.061 ± 0.000	0.064 ± 0.001	0.040 ± 0.001	3.394 ± 0.001	9.215 ± 0.025	33.375 ± 0.001	36.490 ± 0.045	0.232 ± 0.002	0.273 ± 0.001	0.078 ± 0.006	2.397 ± 0.006	26.360 ± 0.024	1.618857 ± 0.001818	13.242 ± 0.012	0.0910 ± 0.0027	0.1222 ± 0.00153
Ahra	0.028 ± 0.001	0.033 ± 0.001	7.971 ± 0.005	0.059 ± 0.002	0.072 ± 0.001	0.041 ± 0.001	3.452 ± 0.009	9.262 ± 0.007	33.570 ± 0.121	35.659 ± 0.093	0.233 ± 0.003	0.270 ± 0.001	0.090 ± 0.003	2.408 ± 0.002	26.163 ± 0.074	1.697 ± 0.003	13.14317 ± 0.037241	0.092 ± 0.0336	0.129 ± 0.0087
III 31 Roger	0.019 ± 0.000	0.023 ± 0.001	7.996 ± 0.008	0.055 ± 0.001	0.063 ± 0.001	0.037 ± 0.001	3.175 ± 0.003	8.394 ± 0.041	31.778 ± 0.095	39.462 ± 0.018	0.243 ± 0.013	0.273 ± 0.001	0.089 ± 0.005	2.190 ± 0.011	26.545 ± 0.051	1.658 ± 0.004	13.333 ± 0.0262	0.0825 ± 0.00213	0.124 ± 0.00166
Hayoul	0.025 ± 0.001	0.026 ± 0.001	8.128 ± 0.011	0.061 ± 0.001	0.066 ± 0.002	0.038 ± 0.001	3.311 ± 0.004	9.607 ± 0.019	30.014 ± 0.011	38.490 ± 0.060	0.255 ± 0.011	0.285 ± 0.001	0.090 ± 0.002	2.498 ± 0.064	26.037 ± 0.030	1.699929 ± 0.04748	13.08217 ± 0.015556	0.095923031 ± 0.002129006457	0.129942433 ± 0.00305194805
Amtalaa Arch	0.019 ± 0.002	0.017 ± 0.001	7.510 ± 0.035	0.078 ± 0.000	0.051 ± 0.001	0.026 ± 0.013	3.515 ± 0.019	8.187 ± 0.020	29.096 ± 0.049	42.762 ± 0.173	0.229 ± 0.018	0.234 ± 0.001	0.090 ± 0.001	2.131 ± 0.008	26.682 ± 0.080	1.632 ± 0.011	13.397 ± 0.042	0.080 ± 0.00103	0.122 ± 0.002571
INRA 2603	0.025 ± 0.000	0.031 ± 0.001	8.197 ± 0.004	0.059 ± 0.000	0.066 ± 0.001	0.038 ± 0.001	4.160 ± 0.011	10.099 ± 0.024	27.752 ± 0.019	38.733 ± 0.056	0.352 ± 0.016	0.271 ± 0.001	0.118 ± 0.002	2.617 ± 0.007	25.528 ± 0.033	1.850 ± 0.005	12.825 ± 0.016	0.1024 ± 0.001982	0.144 ± 0.00294
Aicha Moussa	0.123 ± 0.141	0.027 ± 0.001	8.103 ± 0.060	0.044 ± 0.000	0.075 ± 0.001	0.037 ± 0.001	3.114 ± 0.020	7.589 ± 0.014	36.683 ± 0.046	18.117 ± 0.25547	18.173 ± 0.25378	0.250 ± 0.027	0.177 ± 0.133	1.980 ± 0.010	20.796 ± 0.536	4.257 ± 0.3676	10.453 ± 0.4272	0.095 ± 0.0001	0.4075 ± 0.00860
INRA 2802	0.018 ± 0.001	0.022 ± 0.001	7.942 ± 0.008	0.067 ± 0.000	0.059 ± 0.004	0.035 ± 0.001	3.134 ± 0.005	7.400 ± 0.012	33.451 ± 0.022	39.935 ± 0.067	0.220 ± 0.003	0.237 ± 0.001	0.081 ± 0.010	1.935 ± 0.004	26.928 ± 0.034	1.639 ± 0.004	13.520 ± 0.0172	0.072 ± 0.00105	0.121 ± 0.00258
Rhoul	0.018 ± 0.001	0.022 ± 0.001	6.671 ± 0.006	0.069 ± 0.001	0.055 ± 0.005	0.041 ± 0.003	2.562 ± 0.009	7.685 ± 0.053	34.070 ± 0.047	40.618 ± 0.172	0.186 ± 0.005	0.236 ± 0.001	0.083 ± 0.009	2.008 ± 0.014	27.457 ± 0.091	1.370 ± 0.005	13.786 ± 0.046	0.0731 ± 0.00160	0.099 ± 0.00111
El Qoti Lezreq	0.021 ± 0.001	0.024 ± 0.001	8.238 ± 0.014	0.056 ± 0.001	0.059 ± 0.004	0.039 ± 0.001	3.206 ± 0.015	9.365 ± 0.025	33.853 ± 0.047	35.199 ± 0.117	0.225 ± 0.011	0.271 ± 0.001	0.079 ± 0.004	2.433 ± 0.007	26.139 ± 0.063	1.693 ± 0.006	13.130 ± 0.032	0.093 ± 0.00112	0.1299 ± 0.00217
INRA 2105	0.022 ± 0.001	0.025 ± 0.001	7.562 ± 0.021	0.070 ± 0.002	0.059 ± 0.004	0.036 ± 0.000	2.995 ± 0.006	7.356 ± 0.026	33.161 ± 0.062	40.808 ± 0.023	0.221 ± 0.001	0.239 ± 0.001	0.092 ± 004	1.925 ± 0.007	27.108 ± 0.037	1.57 ± 0.005	13.611 ± 0.019	0.0710 ± 0.00196	0.115 ± 0.00271
Bourqui	0.019 ± 0.002	0.022 ± 0.001	6.815 ± 0.086	0.069 ± 0.001	0.063 ± 0.004	0.040 ± 0.001	3.183 ± 0.032	8.440 ± 0.304	35.387 ± 0.008	36.970 ± 0.725	0.234 ± 0.001	0.246 ± 0.001	0.075 ± 0.003	2.199 ± 0.077	26.932 ± 0.346	1.487 ± 0.018183	13.52517 ± 0.173477	0.0816 ± 0.00223	0.110 ± 0.00105
Bourjassate Noire	0.017 ± 0.001	0.019 ± 0.001	7.833 ± 0.030	0.064 ± 0.001	0.065 ± 0.002	0.041 ± 0.001	3.294 ± 0.005	8.682 ± 0.052	31.894 ± 0.042	38.835 ± 0.098	0.259 ± 0.001	0.234 ± 0.001	0.083 ± 0.001	2.255 ± 0.013	26.470 ± 0.064	1.653 ± 0.005	13.291 ± 0.032	0.085 ± 0.00207	0.124 ± 0.00169
Adroulaniki	0.018 ± 0.001	0.018 ± 0.001	7.886 ± 0.001	0.065 ± 0.001	0.058 ± 0.004	0.037 ± 0.001	3.223 ± 0.006	8.514 ± 0.008	29.245 ± 0.028	41.858 ± 0.037	0.228 ± 0.007	0.247 ± 0.001	0.089 ± 0.004	2.216 ± 0.002	26.539 ± 0.025	1.645 ± 0.003	13.327 ± 0.012	0.083 ± 0.00101	0.123 ± 0.00267

**Table 3:** Variation in fatty acids desaturation and ω-6/ω-3 ratios among cultivars.

Cultivars	ODR	LDR	ω-6/ω-3
Breval Blanca	0.592 ± 0.0833	0.530 ± 0.01	0.885 ± 0.00
Melissosyki	0.584 ± 0.0442	0.469 ± 0.0745	1.131 ± 0.0342
White Adriatic	0.480 ± 0.0538	0.584 ± 0.0577	0.712 ± 0.0723
Kamalata	0.505 ± 0.0387	0.556 ± 0.0865	0.799 ± 0.0156
Lerida	0.529 ± 0.0300	0.542 ± 0.0947	0.842 ± 0.055
Breba Blanca	0.522 ± 0.0525	0.524 ± 0.0532	0.905 ± 0.088
Hafer Jmel	0.573 ± 0.0536	0.512 ± 0.0514	0.954 ± 0.0945
Embar El Khal	0.538 ± 0.037	0.522 ± 0.0967	0.915 ± 0.0317
Ahra	0.547 ± 0.0580	0.515 ± 0.0434	0.941 ± 0.01305
III 31 Roger	0.505 ± 0.0880	0.554 ± 0.0162	0.805 ± 0.05154
Hayoul	0.507 ± 0.0330	0.531 ± 0.085	0.780 ± 0.176
Amtalaa Arch	0.466 ± 0.0284	0.595 ± 0.0779	0.680 ± 0.0283
INRA 2603	0.494 ± 0.0435	0.582 ± 0.0745	0.717 ± 0.0342
Aicha Moussa	0.710 ± 0.002	0.330 ± 0.0998	2.025 ± 0.002
INRA 2802	0.507 ± 0.0336	0.544 ± 0.0753	0.838 ± 0.0326
Rhoul	0.506 ± 0.0369	0.544 ± 0.0783	0.839 ± 0.0276
El Qoti Lezreq	0.551 ± 0.0381	0.510 ± 0.0715	0.9612 ± 0.0397
INRA 2105	0.498 ± 0.0796	0.551 ± 0.0267	0.813 ± 0.0275
Bourqui	0.542 ± 0.0300	0.511 ± 0.0989	0.957 ± 0.011
Bourjassate Noire	0.511 ± 0.0487	0.549 ± 0.0702	0.821 ± 0.0424
Adroulaniki	0.474 ± 0.05	0.589 ± 0.0565	0.699 ± 0.0769

**Table 4:** Analysis of variance (ANOVA) results for oil content (%), fatty acid composition (%), and desaturation pathway ratios across the studied fig seed genotypes.

Variable	Mean	Std	CV (%)	F-Value	*p*-Value
Oil content (%)	27.77	6.45717	23.2507	8.154251	0.000000
C22:0	0.090762	0.027205	29.97435	3.225485	0.000685
C20:1	0.253429	0.03869	15.26648	6.14741	3.93 × 10^−7^
C20:0	1.091019	5.021421	460.2505	2.997145	0.001351
C18:3	37.13293	6.072545	16.35353	5.247349	3.11 × 10^−6^
C18:2	32.31495	2.443698	7.562128	5879.885	5.77 × 10^−66^
C18:1	9.356762	2.72365	29.1089	7458.747	3.91 × 10^−68^
C18:0	3.335619	0.321377	9.634691	1549.288	8.17 × 10^−54^
C17:1	0.047595	0.053607	112.6307	3.067134	0.001096
C17:0	0.063214	0.007155	11.31923	19.50827	3.27 × 10^−15^
C16:1	0.062714	0.008074	12.87443	24.09722	6.73 × 10^−17^
C16:0	7.805857	0.508504	6.514384	514.2585	8.61 × 10^−44^
C15:0	0.024929	0.005696	22.84857	24.0548	6.95 × 10^−17^
C14:0	0.029488	0.033477	113.5289	2.864197	0.002017
TUFA	13.07052	0.879598	6.729634	3.426055	0.000382
TSFA	2.770699	4.521168	163.1778	194.8151	5.04 × 10^−35^
PUFA	26.26821	1.829967	6.966468	3.892632	0.000102
MUFA	2.430125	0.693972	28.55705	5610.327	1.54 × 10^−65^
TSFA/TUFA	0.224631	0.384561	171.1972	56.52292	4.54 × 10^−24^
MUFA/PUFA	0.093914	0.028918	30.7924	56.15978	5.16 × 10^−24^
ω-6/ω-3	13.01706	88.6718	681.1967	1.004605	0.477061
LDR	1.409186	3.96565	281.4144	16,088.53	3.83 × 10^−75^
ODR	1.052425	2.357667	224.0224	6978.209	1.58 × 10^−67^

**Table 6:** Loadings of lipochemical variables on the first three principal components (PC1, PC2, and PC3) in principal component analysis (PCA).

Variables	PC1	PC2	PC3
Oil content	0.005051	0.003635	0.003602
Seeds yield	0.000035	0.029898	0.138464
C14:0	0.000860	0.107673	0.015517
C15:0	0.007966	0.022617	0.202530
C16:0	0.007560	0.015093	0.107982
C16:1	0.005515	0.074496	0.042504
C17:0	0.001142	0.060858	0.107100
C17:1	0.101614	0.004711	0.003983
C18:0	0.002135	0.002384	0.190771
C18:1	0.097336	0.004885	0.001834
C18:2	0.000176	0.034054	0.006347
C18:3	0.006989	0.130976	0.001287
C20:0	0.000386	0.129819	0.051809
C20:1	0.022229	0.003229	0.062323
C22:0	0.000529	0.120594	0.013864
MUFA	0.097632	0.004855	0.001893
PUFA	0.007696	0.130228	0.000575
TSFA	0.102715	0.000601	0.009100
TUFA	0.025287	0.106803	0.007967
MUFA/PUFA	0.100334	0.000056	0.000604
TSFA/TUFA	0.102703	0.000109	0.010479
ODR	0.101551	0.004135	0.006180
LDR	0.100341	0.005895	0.005824
**ω-6/ω-3**	0.102216	0.002395	0.007461

**Table 7:** List of exotic and local genotypes of fig trees (*Ficus carica* L.) used in this study.

Exotic Varieties	Local Genotypes
White Adriatic	Ahra
Breval Blanca	Aicha Moussa
Rhoul	El Qoti Lezreq
Adroulaniki	Hafer Jmel
Kamalata	Lerida
Melissosyki	Embar El Khal
III 31 Roger	INRA 2105
Bourqui	INRA 2603
Amtalaa Arch	INRA 2802
Hayoul	
Breba Blanca	
Bourjassate Noire	

## Data Availability

Data are available upon request.

## Abbreviations

The following abbreviations are used in this manuscript:

ODR	Oleic Desaturation Ratio
LDR	Linoleic Desaturation Ratio
TUFA	Total Unsaturated Fatty Acids
TSFA	Total Saturated Fatty Acids
UFA	Unsaturated Fatty Acids
OA	Oleic Acid
LDL	Low-Density Lipoprotein
WHO	World Health Organization
FAO	Food and Agriculture Organization
GC	Gas Chromatography
FAME	Fatty Acid Methyl Esters
EU	European Union
FTIR-ATR	Fourier-Transform Infrared Spectroscopy-
ANOVA	Analysis of Variance
PCA	Principal Component Analysis
PC	Principal Component
FID	Flame Ionization Detector
